# Correlates and determinants of Early Infant Diagnosis outcomes in North-Central Nigeria

**DOI:** 10.1186/s12981-019-0245-z

**Published:** 2019-09-14

**Authors:** Patrick Dakum, Monday Tola, Nta Iboro, Chukwuemeka A. Okolo, Olachi Anuforom, Christopher Chime, Sam Peters, Jibreel Jumare, Obinna Ogbanufe, Aliyu Ahmad, Nicaise Ndembi

**Affiliations:** 1 Institute of Human Virology Nigeria, Federal Capital Territory, Abuja, Nigeria; 2 Institute of Human Virology, University of Maryland School of Medicine, Baltimore, MD USA; 3 U.S. Centers for Disease Control and Prevention, Federal Capital Territory, Abuja, Nigeria

## Abstract

**Background:**

A negative status following confirmatory Early Infant Diagnosis (EID) is the desired pediatric outcome of prevention of Mother to Child Transmission (PMTCT) programs. EID impacts epidemic control by confirming non-infected HIV-exposed infants (HEIs) and prompting timely initiation of ART in HIV-infected babies which improves treatment outcomes.

**Objectives:**

We explored factors associated with EID outcomes among HEI in North-Central Nigeria.

**Method:**

This is a cross-sectional study using EID data of PMTCT-enrollees matched with results of HEI’s dried blood samples (DBS), processed for DNA-PCR from January 2015 through July 2017. Statistical analyses were done using SPSS version 20.0 to generate frequencies and examine associations, including binomial logistic regression with *p* < 0.05 being statistically significant.

**Results:**

Of 14,448 HEI in this analysis, 51.8% were female and 95% (n = 12,801) were breastfed. The median age of the infants at sample collection was 8 weeks (IQR 6–20), compared to HEI tested after 20 weeks of age, those tested earlier had significantly greater odds of a negative HIV result (≤ 6 weeks: OR = 3.8; 6–8 weeks: OR = 2.1; 8–20 weeks: OR = 1.5) with evidence of a significant linear trend (p < 0.001). Similarly, HEI whose mothers received combination antiretroviral therapy (cART) before (OR = 11.8) or during the index pregnancy (OR = 8.4) had significantly higher odds as compared to those whose mothers did not receive cART. In addition, HEI not breastfed had greater odds of negative HIV result as compared to those breastfed (OR = 1.9).

**Conclusions:**

cART prior to and during pregnancy, earlier age of HEI at EID testing and alternative feeding other than breastfeeding were associated with an increased likelihood of being HIV-negative on EID. Therefore, strategies to scale-up PMTCT services are needed to mitigate the burden of HIV among children.

## Introduction

Early Infant Diagnosis (EID) that yields a negative result is a desired pediatric outcome of programs designed to prevent Mother to-Child Transmission (MTCT) of HIV [1]. For their HIV-infected mothers, staying alive and retained in care with good ART adherence and periodically demonstrated enduring suppression of HIV is the end goal [2]. MTCT in Nigeria accounts for majority of new HIV infections among children worldwide. Worldwide, with about 60,000 new cases reported annually from 2009 to 2012 [3]. In 2015, Nigeria alone was responsible for almost 30% of children newly infected with HIV globally (n = 41,000 [4] of 150,000). Without antiretroviral therapy (ART), more than half of these children will die by 2 years of age [5].

Early Infant Diagnosis utilizes DNA-PCR to isolate viral nucleic acid in HIV-exposed infants (HEI) within 6 weeks of birth [6, 7] and up to 18 months of age [8] thus providing virological basis for entry into lifelong treatment for infected infants. EID impacts epidemic control by confirming non-infected HEIs and prompting timely initiation of ART in HIV-infected babies which improves treatment outcomes [9–11]. Furthermore, the quality of communication from care providers to parents/guardians [3] and the deployment of resources for follow-up of uninfected HEIs [4] are likely to be influenced by knowledge of an EID result.

Despite the importance of EID in mitigating MTCT, its implementation has been challenging in resource-limited settings [12–15], particularly in Nigeria, where only 6.3% and 9% of HEIs received EID in 2014 [16] and 2015 respectively, with only about 20% of those found to be eligible children actually receiving cART [17].

Evaluating current EID programs in relation to where implementation occurs is advocated [18] as this provides context-specific evidence to rethink and adapt current strategies [19]. This study presents data on the prevalence of HIV in HEI and explored the predictors of EID outcomes among HEI who received a DNA-PCR result in the high HIV prevalence settings North-Central Region of Nigeria.

## Material and methods

### Study design

This was a cross-sectional retrospective design utilizing charts of initial DNA PCR testing at 12 weeks of age (or earliest opportunity thereafter) from HEI collected between January 2015 through July 2017.

### Study settings

Institute of Human Virology, Nigeria (IHVN), supports 244 facilities in the Nigerian US President's Emergency Plan for AIDS Relief (PEPFAR) program and all these sites send EID samples to a central molecular diagnostics laboratory for assays.

**Fig. 1:**
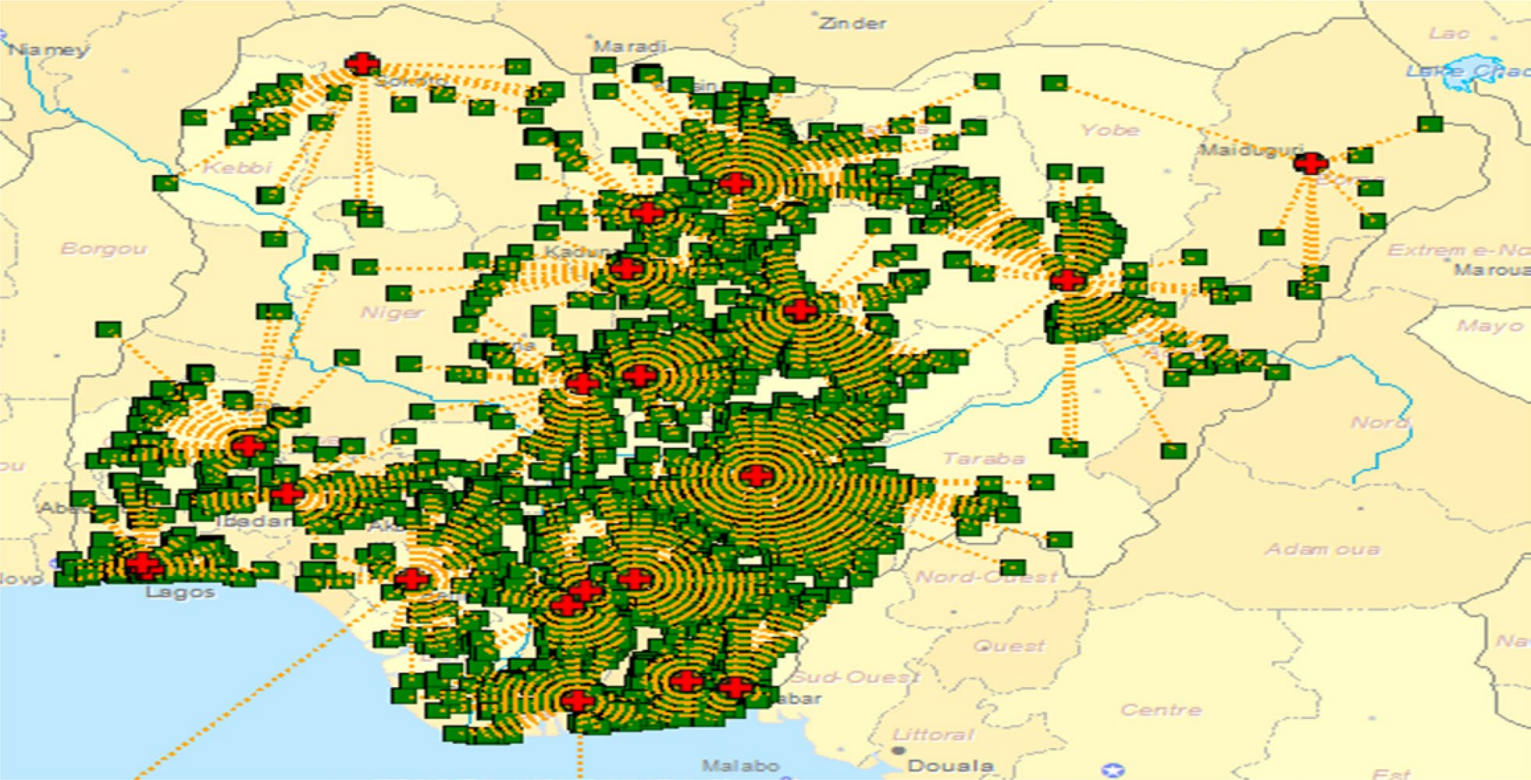
Distribution of facilities providing DBS per geopolitical map of Nigeria, clinics mapped to referral PCR labs for sample referral

Nigeria’s PEPFAR-supported EID program ride on a hub-spoke sample logistics arrangement, whereby peripheral health facilities (those lacking onsite PCR laboratory capacity) have samples collected, batched and delivered to specific public health laboratories for processing. Thereafter, results are dispatched back to these feeder sites (Fig. 1).

Nigeria is organized into six geopolitical zones (GPZ) of which North-Central (NC) including Abuja is one.

Each GPZ is comprised of adjacent states that share similar demography, cultural and political history. Nigeria’s NC is considered her geospatial ‘middle belt’ and has a HIV prevalence rate (2014) of 3.4% similar to the national prevalence rate [20]. Study setting was a molecular diagnostics hub of the Asokoro Laboratory Training Centre (ALTC), located within Nigeria’s Federal Capital Territory (FCT). The laboratory receives and processes dried blood spot (DBS) samples from the FCT and all the facilities in four of seven NC states (Benue, Nasarawa, Kogi and Niger) [21].

### Sample processing

Dried DBS cards (from the facilities) were cut and 1100 µl of specimen pre-extraction reagent (SPEX) was added. The samples were mixed using Eppendorf Thermomixer Comfort (manufactured by Eppendorf AG, Hamburg, Germany) at 56 °C for 10 min at 1000 rpm [17]. Known positive and negative controls were included in each run as per the Manufacturer’s instructions. The samples and controls were then placed in racks and transferred to the Cobas^®^ Ampliprep (CAP) (manufactured by Roche, Mannheim, Germany) where DNA extraction took place for 90 min. Using real-time polymerase chain reaction (PCR), amplification and detection in Cobas^®^ Taqman^®^ (CTM) (manufactured by Roche, Mannheim, Germany) then followed for 3 h 30 min. For positive samples, a repeat assay was carried out. The DBS data processing was done using the Amplilink software version 3.3 [17].

### Data source

De-identified data was retrieved from the laboratory’s Information Management System (LIMS) via export. Information for each data point included: (1) Site Identification number; (2) Date of collection; (3) Location of site by state; (4) Date specimen was received at the processing laboratory; (5) Date of assay (6) Result of Test; (7) Date of dispatch of result; (8) Infant’s age; (9) Infants sex; (10) ART administered to mother; and (11) Breastfeeding status of HEI children.

### Data analysis

Relevant data were cleaned, coded and exported into SPSS version 20.0 (SPSS 2011 IBM Corp, New York, United States) for statistical analysis. We described frequencies, compared associations and performed a binomial logistic regression to ascertain the effects of several factors on the likelihood of infants achieving a negative HIV DNA-1 PCR result. In the model, independent variables explored are sex of HEI (male/female), breastfeeding status (yes/no) and treatment administered to mother: prophylaxis, combination anti-retroviral treatment (cART) during, or cART before index pregnancy. A Cochran–Armitage test of trend was used to see if a linear relationship exists between the time of collection of a DBS sample and the proportion of HEI samples that returned a HIV-positive DNA-PCR result.

## Results

A total of 14,488 DBS sample were analyzed, of which 13,738 (94.8%) returned negative results for HIV DNA-PCR. Median and mean age of HEI at first sample collection (n = 13,646) was 8 weeks (IQR 6–20) and 14.64 weeks (SD = 14.98; 95% CI 0–260) respectively as shown in Table 1.

**Table 1:** Baseline characteristics of mother-HEI pair

Characteristic		Frequency	Percentage (%)
Age (in weeks) of HIV Exposed Infant (HEI) at first dried blood spot collection (DBS): median (IQR)	8.00 (6–20)		
≤ 6 weeks		6000	41.4
6.1–8 weeks		2358	16.3
8.1–20 weeks		2309	15.9
20.1+ weeks		2979	20.6
Missing		842	5.8
Sex of HEI			
Male		6648	45.9
Female		6192	42.7
Missing		1648	11.4
Breastfeeding status of HEI			
Yes		12,801	88.4
No		617	4.2
Missing		1070	7.4
Therapy received by pregnant HIV-infected mother			
No Rx		1010	7
Prophylaxis		159	1.1
cART before pregnancy		7903	54.5
cART during index pregnancy^a^		3651	25.2
Missing		1765	12.2
DNA-PCR result of first DBS			
Negative		13,738	94.8
Positive		750	5.2

^a^Index refers to pregnancy that produced HEI at time of this study

About 41.4% of HEIs had DBS drawn up to 6 weeks, 16.3% between 6 and 8 weeks and 36.5% after 8 weeks. 42.7% (n = 6192) of assayed samples were drawn from female HEI and 88.4% (n = 12,801) of HEI were breastfed (Table 1). Most HIV positive women received combination ART (cART) before the index pregnancy (54.5%, n = 7903), 25.2% (n = 3651) had cART for the first time during the index pregnancy, those who received prophylaxis during labor were 1.1% (n = 159) while 7.0% (n = 1010) received no ART (Table 1).

### Tests of linear relationship

Time of sample collection was at < 6 weeks (n = 6000), 6.1–8 weeks (n = 2358), 8.1–20 weeks (n = 2309), above 20 weeks (n = 2979), and the proportion of positive results returned was 1.9%, 4.0%, 7.1% and 11.4% respectively. The Cochran–Armitage test of trend showed a statistically significant linear trend, p < 0.001, with longer time of collection of DBS associated with a higher proportion of positive DNA-PCR results (Table 2).

**Table 2:** Cross tabulation between age of infant at DBS collection, maternal treatment status and DNA PCR result

Characteristic	Frequency	DNA_PCR of HEI		p-value
		Positive (%)	Negative (%)	
Age of HIV exposed infant (HEI) at first dried blood spot collection (DBS)				< 0.01
≤ 6 weeks	6000	116 (1.9)	5884 (98.1)	
6.1–8 weeks	2358	94 (4)	2264 (96)	
8.1–20 weeks	2309	164 (7.1)	2145 (92.9)	
20.1+ weeks	2979	340 (11.4)	2639 (88.6)	
Total	13,646	714 (5.2)	12,932 (94.8)	
Maternal treatment status				< 0.01
cART	13,478	446 (3.3)	13,032 (96.7)	
Without medication	1010	304 (30.1)	706 (69.9)	
Total	14,488	750 (5.2)	13,738 (94.8)	

### Association of maternal and infant characteristics with EID outcome

To ascertain the associations that age at DBS collection, sex of HEI, breastfeeding status, maternal treatment regimen: prophylaxis, combination anti-retroviral treatment (cART) during, or before index pregnancy had on the likelihood of achieving a negative HIV DNA-PCR result following EID, a predictive model (binomial logistic regression) was performed.

The full model containing all predictors was statistically significant p < 0.001, indicating ability to differentiate those with successful and failed EID outcomes. The model explained between 7.3% of the variance in EID outcomes and correctly classified 94.9% of the cases. 5 variables were statistically significant; breast feeding status of HEI at DBS, age at DBS collection, age of HEI at DBS collection, maternal cART during pregnancy and cART before pregnancy. Controlling for other factors in the model, HEI age at DBS collection of less than or equal to 6 weeks were 4 (3.813) more likely to have HIV negative results when compared to those who had DBS at greater than 20 weeks. Similarly, odds of achieving a negative DNA-PCR result was 2 (2.087) and 1.5 (1.449) for DBS collection at ages 6.1 to 8 weeks and 8.1 to 20 weeks respectively compared to DBS collection at above HEI age above 20 weeks.

cART before pregnancy and during pregnancy were 12 (OR = 11.79) and 8 (OR = 8.349) times more likely respectively, to have HIV negative infants compared to no treatment (see Table 3). Mothers given a prophylaxis regimen during labour had four-fold odds of an infant who returned a negative DNA-PCR result than mothers who received no therapy. Those who did not breastfeed babies at time of DBS had twice the odds of their HEI achieving a negative DNA-PCR result compared to those who breastfed.

**Table 3:** Logistic regression predicting likelihood of negative DNA-PCR based on gender, age at DBS collection, breastfeeding and maternal treatment regimen

Characteristics	EID-HIV negative 1st DNA PCR (N = 13,738)	OR	95% CI		p value
Breastfeeding^a^	12,801	1.94	1.12	3.35	0.018
Maternal treatment					
No maternal treatment	1010	1			0.000
Prophylaxis	159	4.27	2.12	8.62	0.000
cART before pregnancy	7903	11.79	9.41	14.78	0.000
cART during pregnancy	3651	8.35	6.52	10.69	0.000
Age at DBS collection, week					
HEI age at DBS > 20	2979	1			0.000
HEI age at DBS ≤ 6	6000	3.81	2.91	4.99	0.000
HEI age at DBS = 6.1–8	2358	2.09	1.57	2.78	0.000
HEI age at DBS = 8.1–20	2309	1.45	1.14	1.84	0.003
Infant sex (n)^b^%					
Female	6192 (42.7)	0.98	0.81	1.18	0.804
Missing	1648 (11.4%)				

^a^Not breastfed compared to breastfed

^b^Female HEI compared to males

## Discussion

Our study shows that factors like breastfeeding, maternal treatment status (cART or no ART) and age when HEI DBS, can predict the achievement of a negative DNA-PCR result following EID in the High HIV-prevalence settings of North-central Nigeria.

In this study, median age of HEIs at first DBS collection was approximately 8 weeks with only 44% of infants DBS samples taken below 6 weeks of age thereby predisposing the children to a delay in ART initiation for confirmed cases. A longer time of DBS collection and in-effect EID predicted likelihood of HEI being HIV+. For later presentation, it is plausible that symptomatic manifestation of immunologic decline in HIV infection may have prompted mothers/guardians to seek health facility and opportune HEI to EID. A study in Kenya showed that information passed to mother during pregnancy, mothers higher formal education and low experience of stigma could predict on-time (infant ≤ 6 weeks of age) EID [17].

Survival gains of commencing early infant antiretroviral therapy declines when diagnosis is late [22–24] with rapid disease progression and mortality [25] in early life. Timely definitive diagnosis is critical in allowing early initiation of life-saving ART [26] as demonstrated in the children with HIV early antiretroviral (CHER) study [27], which reported a 76% and 75% aversion of early infant mortality and HIV progression respectively [28]. Performance of Nigeria’s EID logistics is affecting EID outcomes.

Breastfeeding is important for child survival [29, 30] and consistent with other studies in Nigeria [28, 31], was common among study mother-HEI pairs 88.9% (n = 3232). Mothers living with HIV, especially in LMIC, should breastfeed for 12 months and may continue breastfeeding for up to 24 months or longer, while being fully supported for ART adherence [32]. This is to help fight against malnutrition and death resulting from cholera due to poor hygiene. Breastfeeding should only stop once a nutritionally adequate and safe diet without breast milk can be provided [32]. Breastfeeding by HIV positive mothers was a significant predictor of HEI outcome in this study and is linked to continuing risk of postnatal infection [33], with documented MTCT rate of 13% at six weeks rising to 23% at the end of breastfeeding [34]. Data on type (exclusive or mixed), duration [35] and frequency of breastfeeding, mother’s immunological staging (HIV viral load/CD4+) and HEI testing after a 6-week window period following stop of breastfeeding [12] is required to further investigate this and is recommended.

Majority (54.5%) of HIV positive mothers were already on cART before the index pregnancy, whilst almost a third received cART for the first time. Maternal prophylaxis and cART were significant predictors of EID outcomes. Exponential rises in the chance of achieving a negative DNA-PCR result for HEI was related to the timing of cART with best results if mother started cART prior to pregnancy. This finding stands unadjusted for Mother’s cART adherence, duration of therapy or immunological status, and for the method of delivery (vaginal or operative) or timing of EID testing. It is thus deduced that aside these and other factors, substantial program-wide benefit of averting MTCT is achieved if cART for mother is ensured.

This speaks to the present MTCT issue in Nigeria where only 29% of HIV-positive pregnant women received ART [36] in 2014. Hence, as a strategy to mitigate the growing population of the 380,000 HIV-positive children residing in-country [20, 37], prevention of MTCT and EID programs in Nigeria need to urgently scale-up ART coverage for HIV infected females of reproductive age and mothers.

Nigeria adopted the WHO ‘Test and Treat’ policy in June 2016 [38] to accelerate early and universal placement on ART for all HIV positive persons [2]. In prevention of MTCT this translates to starting HIV positive pregnant women early on lifelong cART irrespective of clinical or immunological criteria (option B+) [39]. The difference in odds between the earlier placement of HIV positive mothers (previous vs index pregnancy) supports the notion that optimization of favorable pediatric outcomes is achieved at earliest cART initiation of all peripartum HIV-infected women [40].

## Limitations

This study was limited by the extent of variables utilized, due to non-availability of others such as baseline maternal viral load or clinical staging and follow-up HEI testing on cessation of breastfeeding. This limited our ability to explore the role of other characteristics that may be linked to EID outcomes, in addition to possible incomplete control of confounding for the associations explored. Another limitation is potential selection bias due to low EID rates among PMTCT clients which may affect generalizability of the findings. Another major challenge is the issue of missing data as request forms from the clinics are not completely filled. Clinicians needs to be aware of the importance of properly filling request forms, to enable us have a good picture of the program during evaluation.

## Conclusion

This study showed that provision of cART for HIV infected mothers prior to and during pregnancy is associated with an increased likelihood of achieving a negative EID result. Overall program wide benefit can be optimized if lifelong cART coverage is increased for HIV positive pregnant women.

## Data Availability

The datasets during and/or analyzed during the current study available from the corresponding author on reasonable request.
